# Five-Year Outcomes of Endovascular Aneurysm Repair (EVAR) Performed Using a Polymer-Based Aortic Endograft System

**DOI:** 10.7759/cureus.93970

**Published:** 2025-10-06

**Authors:** Faris Saleh, Ibraheem Obaidat, Prashant Neupane, Mohammed Ashrafi, Hiren Patel

**Affiliations:** 1 Vascular Surgery, Manchester University NHS Foundation Trust, Manchester, GBR; 2 Vascular Surgery, Manchester Royal Infirmary, Manchester, GBR; 3 General Surgery, School of Medicine, University of Jordan, Amman, JOR

**Keywords:** evar, infrarenal aaas, ovation polymer based endograft, technical success, vascular surgery

## Abstract

Background

Endovascular aneurysm repair (EVAR) is a widely used, minimally invasive alternative management option to open surgical repair for infrarenal abdominal aortic aneurysms (infrarenal AAAs). However, standard stent grafts have their challenges for complex aortic anatomies. The Ovation Abdominal Stent Graft System (Endologix Inc., Santa Rosa, CA), which is a polymer-filled sealing ring endograft, has emerged as a promising solution for patients with challenging anatomical features. This study aims to evaluate the Technical success, effectiveness, and long-term outcomes, including reintervention and survival, of the Ovation system in treating complex infrarenal AAAs.

Methods

We conducted a single-centre, retrospective observational study of 103 patients with anatomically challenging infrarenal AAAs treated with the Ovation stent graft between January 2015 and November 2017 in a UK-based tertiary centre. Anatomical parameters included short necks, severe angulation (>60°), significant thrombus or calcification (>180 degree of the wall diameter), non-cylindrical neck shapes, or an iliac access diameter <6 mm. Outcomes assessed included technical procedural success, Endoleak incidence, Conversion to open, reintervention rates, 30-day aneurysm-related mortality, and all-cause mortality over a 5-year follow-up.

Results

A total of 103 patients underwent EVAR using the Ovation stent graft between January 2015 and November 2017, with a 5-year follow-up. All patients had at least one adverse anatomical feature, including short necks, significant thrombus or calcification, severe neck angulation, or narrow iliac access. The cohort was predominantly male, comprising 80 patients (77.7%) with a mean age of 76.4 years (SD = 6.67). Technical success was achieved in 97% of cases, with Type I endoleak observed intraoperatively in 3 patients (2.9%). Intraoperative complications occurred in 4.8% of patients, with no aneurysm rupture or conversions to open surgery. The 30-day aneurysm-related mortality was 0%, and the overall reintervention rate over five years was 9.7%. The five-year all-cause mortality rate was 16.5%.

Conclusions

The Ovation polymer-based endograft demonstrates excellent technical success and favourable long-term outcomes in patients with infrarenal AAA with unfavourable anatomy, including low rates of type I endoleaks, reintervention, and aneurysm-related mortality. These findings support its use as a durable and effective EVAR solution in anatomically challenging cases beyond the standard IFU. Further multicentre prospective studies are warranted to confirm these promising results.

## Introduction

Endovascular aneurysm repair (EVAR) has gained significant acceptance due to its effectiveness and its less invasive alternative to the conventional open surgical approach for treating most infrarenal abdominal aortic aneurysms (infrarenal AAAs) [1-3,4]. Nevertheless, not all AAAs are amenable to standard stent-frame EVAR; for instance, those with adverse anatomical features, such as hostile neck sealing zones or tortuosity, and those complicated by thrombus and inflammation [5-7]. In recent years, more focused attention has been paid to polymer-based technology as an alternative approach to sealing AAA, which is designed to accommodate a wider range of aortic morphology [8].

Polymer-based aortic endografts such as the Ovation Abdominal Stent Graft System (Endologix Inc., Santa Rosa, CA), also referred to as O-ring EVAR, have been developed to stent infrarenal AAAs with more complex anatomy outside the traditional stent graft instructions for use (IFU) range, including infrarenal neck angulation ≥60, neck length ≤15mm, infrarenal diameter 18-32mm, and iliac access diameter ≤6mm. It incorporates an improved seal mechanism to enhance patient safety, which can decrease the likelihood of endoleak and sac enlargement [9-11]. The selection of polyethylene glycol-based polymer in the Ovation system was based on its non-thrombogenic features and its biocompatibility properties [8, 12, 13].

The polymer had been developed to be soluble and non-embolic in the event of unintentional leakage into the vasculature after mixing [8]. The Ovation system has been associated with a lower likelihood of type I endoleak related to endograft migration and aortic neck dilatation, as the graft has a unique fixation mechanism that exerts less continuous radial force [14]. Several studies have reported that this type of device is effective and safe for use in the short and medium term in managing infrarenal AAAs with challenging anatomical characteristics [1,15]. However, there is a lack of literature examining the long-term outcomes and durability of this device. Thus, this study aims to evaluate the long-term survival, complications, and durability of EVAR using the Ovation stent graft system in managing infrarenal AAAs.

## Materials and methods

Study design and participants

This was a single-centre retrospective observational study conducted at a regional tertiary centre (Wythenshawe Hospital and Manchester Royal Infirmary, Manchester, UK). Institutional review board approval and patient consent were not required, as this was a retrospective study of prospectively collected information on an electronic database regarding outcomes following procedures and a review of patient case notes. There is no identifiable patient data presented, and therefore, there is no way to identify individual patients; consequently, patient consent was not required. This work has been reported in line with the Strengthening the Reporting of Observational Studies in Epidemiology (STROBE) criteria [16].

Although the indication for IFU, for the Alto graft is for proximal landing zone of 7mm, conicity less than 10%, aortic angle less than 60 and lack of significant thrombus or calcification [17], patients eligible for inclusion were those diagnosed with infrarenal AAAs who had adverse anatomical features and underwent endovascular repair using EVAR with an Endologix Ovation endograft, between January 2015 and November 2017 and followed up for a five-year duration. Following routine cardiopulmonary exercise testing, the infrarenal AAAs cases were discussed in a multidisciplinary team meeting before settling on definitive treatment methods. The use of the Ovation endograft has been considered for patients exhibiting atypical aneurysmal anatomical characteristics. The characteristics of atypical aneurysmal anatomy encompass various factors, such as an infrarenal neck angulation of more than 60°, a neck length less than 15mm, an infrarenal neck diameter falling below 18mm or exceeding 32mm, an iliac access diameter less than 6mm, a substantial presence of thrombus or calcific burden at the aortic neck( >180 degree of the wall diameter), or an unfavourable neck shape. The implantation of all stent grafts was performed collaboratively by a consultant vascular surgeon and a Consultant Interventional Radiologist.

Patients excluded from the study were those who had EVAR using other devices, those treated with chimney EVAR, individuals who received Ovation stents for aneurysms without unfavourable anatomical features, and patients treated with fenestrated stent grafts.

Data collection

Retrospective data extraction was conducted from hospital-based electronic patient records (Chameleon Electronic Patient Record) and imaging software (Patient Archiving and Communication Software - PACS, GE systems, Pollards Wood, UK. The collected data included demographics (age and sex), comorbidities (hypertension, ischaemic heart disease (IHD), hypercholesterolemia, chronic obstructive pulmonary disease (COPD), chronic kidney disease (CKD), diabetes mellitus (DM), and smoking status), anatomical characteristics of the infrarenal AAAs (aneurysm diameter, proximal neck diameter, length and shape, angulation, presence of thrombus and calcification, and iliac access diameter), operational characteristics (date of operation, anaesthesia, vascular access, and intraoperative complications), and follow-up variables (postoperatively, reintervention, complications, and death). Measurements about the anatomy of the infrarenal AAAs were collected using pre- and postoperative computed tomography (CT) angiography, duplex imaging, and follow-up assessments. The hospital conducted regular postoperative follow-ups for all patients who had EVAR. These follow-ups consisted of arterial phase CT scans at three months and one year following the procedure, followed by yearly duplex ultrasound examinations. Further Investigations were conducted using CT scans and contrast-enhanced duplex to examine any potential anomalies detected throughout the follow-up period.

Outcome measures

The primary outcome measure was the all-cause mortality rate over a five-year follow-up period. The secondary outcomes were 30-day aneurysm-related mortality and the primary success of the procedure (characterised as the absence of type 1 or type 3 endoleak intra- or peri-operatively), as well as the need for reintervention due to complications. Technical procedural success was defined as successful navigation, delivery, and deployment to the target site of the main body and iliac limbs and the absence of type 1 endoleak.

Statistical analysis

Continuous variables were described as means and standard deviation (SD), while categorical variables were presented as frequencies and percentages (%). Cases with missing all outcome measures were excluded. Missing data for all study variables were reported and excluded from the analysis. All statistical analyses were conducted using JASP software version 0.19.3 (JASP, University of Amsterdam, Amsterdam, The Netherlands). Statistical analyses were considered significant when the p-value was less than 0.05.

## Results

A total of 103 patients underwent EVAR between January 2015 and November 2017 using the Ovation stent graft and had a follow-up period of five years. All the patients included in this study have at least one adverse anatomical feature, which includes a short neck, significant thrombus load, significant calcification, or angulated neck. The majority of the cases were elective procedures; however, 5 out of 103 (4.85%) of the cases were urgent, involving either rupture or symptomatic aneurysms.

There were 80 (77.7%) male patients and 23 (22.3%) female patients. The mean age was 76.4 (SD 6.67). The patients' comorbidities and smoking status are illustrated in Table 1.

**Table 1:** Comorbodities and smoking status

Comorbodities and smoking status	Number of patients (%)
Diabetes	12 patients (11.7%)
Hypertension	89 patients (85.4%)
Hypercholesterolemia	44 patients (42.7%)
Active smoker	24 patients (23.3%)
Ischemic heart disease	51 patients (49.5%)
Chronic kidney disease	20 patients (19.4%)
Chronic obstructive pulmonary disease	41 patients (39.8%)

The mean aneurysmal diameter was 6.2 (SD 0.83). The mean diameter for the neck was 23.8 mm (SD 3.15), although 75 patients (72.8%) had a neck length of 15 mm or more, and only 17 patients (17.5%) had a straight (cylinder) neck shape. The rest of the patients' neck shapes included other adverse features described in Table 2. A total of 30 patients (29.12%) had significant calcifications in the neck of the aorta, and 40 patients (38.8%) had a significant thrombus load at the proximal neck of the aorta. The infrarenal angle was more than 60 degrees in 20 patients (19.4%). Open access was utilised in 75 patients (72.8%). The external iliac artery diameter was less than 6mm in five patients (4.85%).

**Table 2:** Neck shapes

Shape of neck	Number (%)
Conical	75 (72.8%)
Reversed Conical	7 (6.8%)
Barrel-shaped	2 (1.9%)
Saccular (localised bulge)	1 (0.97%)
Straight	17 (17.5%)

Technical procedural success was observed in 100 cases (97%). Three cases had type 1 endoleak (one case was type 1a, which required a Palmaz stent as an additional procedure). There were no cases of intraoperative aneurysm rupture or conversion to open surgery. However, there were five intraoperative complications (4.8%), one related to the closure device failure, one anaphylactic reaction during the procedure, one patient required chimney stents to both renal arteries due to partial coverage of the renal arteries orifices, and one case had a twist of the unsupported part of the stent graft, which required proximal extension, and the last case had migration of the iliac stents which required limb re-lining. A total of 13 patients (12.6%) had type 2 endoleak identified intraoperatively; however, only two patients (1.94%) had persistent endoleak in the follow-up scans. The re-intervention rate was seen in 10 patients (9.7%); i.e., 10 patients had another procedure during the five-year follow-up period. 

The 30-day aneurysm-related mortality rate was zero, and all-cause mortality (non-aneurysm-related) was seen in 17 cases (16.5%) at the end of the follow-up period. Due to the low number of intraoperative complications and type 1 endoleaks, it was not possible to draw any relation with any of the potential risks that were studied, including adverse neck features (short neck, calcification, non-cylindrical, angulated, and significant thrombus load) or small external iliac diameter.

## Discussion

This study presents a comprehensive five-year analysis of the Ovation Abdominal Stent Graft System's long-term effectiveness in managing infrarenal AAAs in anatomically challenging features. The high primary success rate and the reduction in aneurysm diameter over two years demonstrate the potential utility of this polymer-based stent in challenging anatomical conditions. The reintervention rate was seen in 10 cases (9.7%), with only three cases of developing type I endoleak, and no patients had type III endoleak. Although the overall all-cause mortality rate was 17/103 (16.5%), non-aneurysm-related mortality was nil. These findings reinforce the potential of the Ovation system in managing complex infrarenal AAAs and its durability over time, and as a treatment option in the medium to long term for patients with infrarenal AAAs who are unsuitable for traditional stent-grafts due to complex anatomical characteristics outside of the IFU.

In this study, we found that no patient had graft migrations over a five-year period, which is consistent with an earlier study that demonstrated there were no cases of graft migrations in a mid-term experience of the five-year follow-up period [18]. Several retrospective studies have demonstrated favourable midterm results using the Ovation stent graft in real-world scenarios, including patients with anatomical characteristics that range from acceptable to challenging [15,19,20]. Nevertheless, this finding contrasts with a meta-analysis, which reported graft migration in nine patients (8.7%) associated with conventional self-expanding stent-grafts, which provide consistent radial pressures on the aorta wall, unlike the Ovation stent graft [21].

According to the AAA Practice Guidelines established by the Society for Vascular Surgery, it is vital to maintain frequent and constant monitoring to detect endoleaks after EVAR, explicitly identifying type Ia endoleaks [22]. The technical challenges associated with complications of the proximal neck are especially noteworthy [23]. Among the cohort of 103 patients, three developed a type I endoleak requiring intraoperative adjunctive intervention. In addition, type II endoleaks were identified intraoperatively in 13 patients (12.6%); however, only two patients demonstrated persistence of the endoleak on follow-up imaging. As shown in a previous study, the incidence of type I or type III endoleak and secondary intervention is relatively low [20], showing a favourable comparison to similar studies conducted on endograft trials [24,25], which support the findings in this study.

Turney et al. identified that type IA endoleak was the prevailing indication for explantation in a large series of EVAR stent-graft explants. In addition, they observed that the enlargement of the neck region was often associated with the migration of the device and the occurrence of type IA endoleak [26]. Several studies have shown comparable favourable findings, identifying type IA endoleak as the primary indication for open conversion [27,28]. In our study, although three patients had a type-1 endoleak, none experienced rupture or required conversion to open surgery, underscoring the procedural safety and durability of the Ovation stent graft. Notably, the primary technical success rate was seen in 100 cases (97%), which is comparable to the 100% reported by Mehta et al. [1]. We achieved a 100% 30-day survival, mirroring the low 30-day major adverse event rate of 2.5% described in their pivotal trial. Our study also demonstrated no aneurysm-related mortality, aligning with the 0.6% AAA-related mortality at one year in their series and the overall mortality being 16.5%, primarily attributable to non-aneurysmal causes. Similarly, no cases of stent graft migration consistent with their reported outcomes.

A total of 5 out of 103 patients (4.8%) subsequently developed intraoperative complications, with 10 patients (9.7%) requiring an additional procedure during the five-year follow-up period. None of the patients experienced graft migration. These results are consistent with previous studies in which no incidents of stent graft migration or type I or III endoleaks were reported throughout follow-up [1,15]. The findings of these earlier studies are also in line with larger Ovation-based trials, further supporting the effectiveness of this procedure [1,9,19,29] and aligning with the outcomes observed in our study. Future analyses could further explore the efficacy of these grafts in female patients, whose iliac arteries are generally smaller in diameter compared to males and may therefore limit the suitability of conventional stent grafts.

The strength point of our study is the considerable sample size, and it provides long-term findings that may be extrapolated to a diverse array of aortic anatomical variations. One of the limitations of this research was that it was a single-centre retrospective study and the absence of a control group, which resulted from the non-randomized design. Hence, comparing trial outcomes with those seen in other studies on EVAR or surgical interventions may be subject to confounding due to variations in patient demographics, trial methodologies, stent graft efficacy, or other relevant variables.

## Conclusions

This paper presents findings from a comprehensive analysis of a substantial dataset, which establishes the efficacy and safety of the Ovation polymer-based aortic endograft device for delivering favourable outcomes in a long-term period as a reliable therapy option for patients diagnosed with infrarenal AAAs. It also shows that the Ovation stent graft is a valid option for the management of infrarenal aneurysms in patients with adverse anatomical features.
